# Expression of interferon-stimulated genes, but not polymorphisms in the interferon α/β receptor 2 gene, is associated with coronavirus disease 2019 mortality

**DOI:** 10.1016/j.heliyon.2024.e39002

**Published:** 2024-10-05

**Authors:** Berliana Hamidah, Cennikon Pakpahan, Laksmi Wulandari, Damayanti Tinduh, Tri Wibawa, Cita Rosita Sigit Prakoeswa, Delvac Oceandy

**Affiliations:** a Department of Biomedical Science, Faculty of Medicine, Universitas Airlangga, Surabaya, Indonesia; b Department of Pulmonology and Respiratory Medicine, Faculty of Medicine, Universitas Airlangga/Dr Soetomo General Academic Hospital, Surabaya, Indonesia; c Department of Physical Medicine and Rehabilitation, Faculty of Medicine, Universitas Airlangga/Dr Soetomo General Academic Hospital, Surabaya, Indonesia; d Department of Microbiology, Faculty of Medicine, Public Health, and Nursing, Universitas Gadjah Mada, Yogyakarta, Indonesia; e Department of Dermatology, Venerology and Aesthetics, Faculty of Medicine, Universitas Airlangga / Dr. Soetomo General Academic Hospital, Surabaya, Indonesia; f Division of Cardiovascular Sciences, Faculty of Biology, Medicine and Health, University of Manchester, Manchester, United Kingdom

**Keywords:** COVID-19, Interferon, Immune response, Interferon receptor, IFNAR2, Interferon type I, Interferon-stimulated genes, Polymorphism

## Abstract

Excessive inflammatory response is a hallmark of severe COVID-19. This study investigated the associations between interferon-stimulated genes (ISGs) expression, genetic variation in the interferon α/β receptor 2 (IFNAR2) gene, and COVID-19 mortality.

We investigated 67 patients with moderate-to-severe COVID-19. Of them, 22 patients (32.8 %) died because of COVID-19. We examined the expression of ISGs in total RNA of peripheral whole blood. We observed a significant increase in the expression of all ISGs examined in non-surviving patients, indicating a heightened interferon type I signaling activation in non-survived patients. Subsequently, we analyzed whether the increase in ISGs expression was correlated with polymorphism within the *IFNAR2* gene. Intriguingly, no significant association was observed between *IFNAR2* gene polymorphism and COVID-19 mortality. Similarly, no association was noted between the IFNAR2 and ISGs expression levels.

Overall, our data showed that higher ISGs expression, which presumably indicates heightened interferon type I activation, is associated with COVID-19 mortality.

## 1 Introduction

The interferon signaling pathway is critical in the regulation of both innate and adaptive immune responses against pathogens, particularly viruses [1]. Interferons mediate their functions by binding to their receptors and activating a downstream signaling cascade that eventually induces interferon-stimulated genes (ISGs) expression [2]. ISGs eventually mediate the antiviral, antiproliferative, and immunomodulatory effects of interferons [3]. Of the three types of interferon signaling (types I–III), type I interferon is widely known to play crucial roles in various infectious diseases caused by viruses, bacteria, and parasites [1]. For example, reduced type I interferon signaling activation leads to severe dengue virus infection [4], whereas impaired interferon type I signaling is also present in patients with chronic hepatitis [5]. More recently, coronavirus disease 2019 (COVID-19) severity has been associated with the interferon signaling pathway [6], as higher interferon levels, in particular in lower respiratory tract, are associated with disease severity and increased mortality in COVID-19 [7,8].

An excessive inflammatory response is strongly correlated with poor COVID-19 prognosis [9]. The inability of the immune response to clear the virus may lead to the induction of inflammation, which eventually causes multiple detrimental effects in various organs in patients with severe COVID-19 [10,11]. Several studies have shown that the interferon type I response is differently regulated during different stages of COVID-19. At the early stage of this disease, interferon type I responses were lower than those mediated by other cytokines, including interleukin 6 (IL-6) and tumor necrosis factor alpha (TNF-α) [12]. However, the lower level of interferon induction in the upper respiratory tract may compromise the antiviral capacity of the immune response, thereby facilitating virus survival and entry into the lower respiratory tract. Subsequently, this can lead to an exaggerated inflammatory response characterized by elevated interferon levels, alongside other cytokines and chemokines in the lower respiratory tract. Consequently, severe COVID-19 cases are frequently marked by a low interferon response at the early stage in the upper respiratory tract, followed by an excessive interferon response in the later stages within the lower respiratory tract [13,14].

Recent genome-wide association studies (GWAS), involving 2244 patients with COVID-19, have revealed an association between single nucleotide polymorphism (SNP) within the interferon α/β receptor 2 (IFNAR2) gene and COVID-19 severity [15]. The *IFNAR2* gene encodes the subunit of the type I interferon receptor and, in conjunction with IFNAR1, forms the heterodimeric type I interferon receptor. This receptor interacts with and activates Janus kinase (JAK) and tyrosine kinase 2 (TYK2) upon stimulation [16]. Specifically, the study identified a link between an SNP within the human *IFNAR2* gene (rs2236757 polymorphism) and COVID-19 severity [15]. Interestingly, in a separate study, Zhang et al. demonstrated a correlation between severe COVID-19 cases and defects in genes associated with the type I interferon signaling pathway [17].

Despite the strong association between IFNAR2 polymorphism (rs2236757) and COVID-19 severity, it remains unclear whether this polymorphism interferes with interferon signaling and immune response in patients with COVID-19. To address this question, in this study, we investigated whether the IFNAR2 polymorphism was associated with interferon signaling in patients with COVID-19 by analyzing the induction of ISGs expression. Moreover, we investigated whether this genetic variation and the level of ISGs expression were correlated with patient survival.

## 2 Materials and methods

### 2.1 Study design and patients

This cross-sectional study focused on patients with moderate and severe confirmed COVID-19. It was conducted between two periods, first between June 22 and August 19, 2020, and then between September and October 2021. We performed power analysis to determine sample size based on the finding that expressions of interferon stimulated genes (IRF-3 and IRF-7) were differently regulated by ∼30 % in COVID-19 patients with a standard deviation of ∼20 % of the mean [18]. Power analysis suggested that to achieve a statistical power of 80 % (α = 0.05) a minimum number of 22 patients per group was required. Since this study compared ISGs expression between survived and non-survived patients, we aimed at recruiting a minimum number of 22 participants in each group during the period of the study. We recruited a total of 67 patients; 45 of them survived and recovered from COVID-19, whereas 22 patients died due to COVID-19 or its complications. All of the patients fulfilled the criteria of inclusion: i) have moderate or severe COVID-19, and ii) have not been vaccinated for COVID-19 at the time of admission. All patients were diagnosed with the SARS-CoV-2 infection using a nucleic acid amplification test (NAAT). They were hospitalized in Dr. Soetomo General Academic Hospital, Surabaya, Indonesia. Patients were categorized as having moderate or severe COVID-19 according to the WHO Guideline for COVID-19 management [19]. This study received ethical approval from the Local Ethics Committee of Dr. Soetomo General Academic Hospital, Surabaya, Indonesia (0254/KEPK/IX/2021). All patients agreed to participate and signed the informed consent form for this study.

### 2.2 Sample collections

DNA and RNA were isolated from peripheral whole blood samples. Heparinized peripheral blood samples were collected at the time of admission and stored in an −80 °C freezer. NAAT for COVID-19 diagnosis, complete blood count, and C-reactive protein (CRP) test were performed in Dr. Soetomo General Academic Hospital, Surabaya, Indonesia, as part of the standard procedure for patients with COVID-19. DNA and RNA isolation, genotyping, and mRNA analyses were performed at the Institute of Tropical Disease, Universitas Airlangga, Surabaya, Indonesia.

DNA extraction was performed using the QIAamp Blood DNA Midi Kit (Qiagen) as previously described [20]. Total RNA was extracted using the QIAzol reagent (Qiagen) followed by purification using RNeasy spin column (RNeasy Mini Kit, Qiagen) following the manufacturer's instructions. DNA and total RNA extraction procedures were performed in a biosafety level 3 laboratory. DNA and total RNA yields were determined using a microvolume spectrophotometer (NanoDrop Lite, Thermo Fisher Scientific).

### 2.3 IFNAR2 polymorphism detection

The IFNAR2 polymorphism (rs2236757) was detected using a TaqMan SNP Genotyping Assay for humans (Applied Biosystems) following the manufacturer's instructions. Genotyping was performed using the 7500 Fast Real-Time Polymerase Chain Reaction (RT-PCR) System (Applied Biosystems) with VIC and FAM fluorescence to report allelic discrimination. To construct the allelic discrimination plot, the 7500 software v2.3 (Life Technologies, Applied Biosystems) was employed.

### 2.4 Analysis of IFNAR1, IFNAR2, and ISGs mRNA levels

Total RNA (2 μg) was reverse transcribed to cDNA using a High-Capacity cDNA Reverse Transcription Kit (Applied Biosystems) according to the manufacturer's protocol. To determine the mRNA levels of IFNAR1, IFNAR2, and ISGs, including IFITM1, TRIM5, ISG15, viperin, IFIT1, MX1, and CH25H, the QuantStudio 1 RT-PCR System (Applied Biosystems) with SYBR Green reporter was used. Expression of housekeeping genes (ACTB and RPS13) was detected as an internal loading control. All of the primer sequences were obtained from the Primer Bank database (https://pga.mgh.harvard.edu/primerbank/). All primers in this resource were specifically designed for quantitative mRNA detection [21]. The primer sequences are presented in Table 1.

**Table 1:** List of primers used and their sequences.

Gene name	Forward primer (5′–3′)	Reverse primer (5′–3′)
IFNAR1	ATTTACACCATTTCGCAAAGCTC	TCCAAAGCCCACATAACACTATC
IFNAR2	ACCACTCCATTGTACCAACTCA	TGTGCTTCTCCACTCATCTGT
IFIT1	AGAAGCAGGCAATCACAGAAAA	CTGAAACCGACCATAGTGGAAAT
IFITM1	CCAAGGTCCACCGTGATTAAC	ACCAGTTCAAGAAGAGGGTGTT
ISG15	TGGACAAATGCGACGAACCTC	TCAGCCGTACCTCGTAGGTG
CH25H	GCTGGCAACGCAGTATATGAG	CGAGCAGTGTGACGTTCATC
TRIM5	AAGTCCATGCTAGACAAAGGAGA	GTTGGCTACATGCCGATTAGG
Viperin	TTGGACATTCTCGCTATCTCCT	AGTGCTTTGATCTGTTCCGTC
MX1	GGTGGTCCCCAGTAATGTGG	CGTCAAGATTCCGATGGTCCT
ACTB	CATGTACGTTGCTATCCAGGC	CTCCTTAATGTCACGCACGAT
RPS13	TCCCAGTCGGCTTTACCCTAT	CAGGATTACACCGATCTGTGAAG

mRNA expression levels were determined using delta Ct values against the Ct values of the housekeeping genes. The optimum housekeeping gene to be used as a reference was determined using NormFinder ver.20 (https://www.moma.dk/software/normfinder) [22]. We analyzed the expression of ACTB and RPS13 as reference genes. Analysis using NormFinder version 20 suggested that RPS13 displayed better performance when used as a reference gene. Thus, all gene expressions were normalized to the RPS13 level. We calculated relative mRNA expression between groups using 2^−ΔΔCt^ method as described elsewhere [23].

### 2.5 Data analysis

Data were presented as means ± SEM, unless stated otherwise. The association between categorical variables in the patients' demographic data was analyzed using the chi-square or Fisher's exact test. For numerical variables, we used the Shapiro-Wilk test to analyse the normal or lognormal distribution of the data. Comparisons of inflammatory markers and ISGs expressions between the surviving and non-surviving groups were performed using either independent t-tests if the data was normally distributed or Mann-Whitney *U* test if the data was not normally distributed. Analysis of IFNAR1/2 and ISGs expression according to IFNAR2 rs2236757 genotypes was performed using one-way analysis of variance, or Kruskal-Wallis test, depending on whether the data was normally distributed or not, followed by multiple comparison tests. To assess the association between patients' characteristics, comorbidities, corticosteroid treatment, and ISGs expressions we conducted multivariate regression analysis. Associations between IFNAR2 rs2236757 genotypes, IFNAR2 mRNA level, IFNAR1 mRNA level, and ISGs expressions were also analyzed using multivariate regression analysis. A *P* value of <0.05 was considered statistically significant. Statistical analyses were performed using SPSS software version 25 (IBM, Armonk, NY, USA) or GraphPad Prism version 9 (GraphPad Software, LLC).

## 3 Results

### 3.1 Patients’ characteristics

We investigated 67 patients with confirmed COVID-19 who were admitted to Dr. Soetomo General Academic Hospital between two periods, first between June and August 2020 and between September and October 2021. All patients were not vaccinated at the time of admission. All of the patients included in this study had moderate-to-severe COVID-19 based on the WHO criteria [19]. All patients received standard supportive therapy according to treatment guideline [19,24]. A total of 28 patients with severe COVID-19 received corticosteroid therapy (dexamethasone 6 mg/day). The criteria of corticosteroid treatment were based on NIH guidelines for COVID-19 treatment [24]. Of total 67 patients included in this study, 45 (67.2 %) survived and recovered completely from the disease, whereas 22 (32.8 %) died because of COVID-19 or its complications. In this study, our analyses were conducted according to patients’ outcomes, comparing the parameters between recovered (survived) and non-recovered (died) patients.

Demographic characteristics of the patients are described in Table 2. No significant difference in age was observed between recovered and non-recovered patients. Meanwhile, male patients had a higher proportion of death than their female counterparts (39.1 % vs. 19 %); however, it did not reach statistical significance. Moreover, there was no significant association between the number of comorbidities and COVID-19 survival; however, the trend showed a higher proportion of non-survival in patients with one or more comorbidities (33%–40 % fatality) than that of participants without comorbidities (18 % fatality).

**Table 2:** Demographic and baseline characteristics of the study participants.

	All patients (n = 67)	Survived (n = 45, 67.2 %)	Non-survived (n = 22, 32.8 %)	P value
**Age** (mean ± SD)	50.1 ± 10.6	50.0 ± 12.1	50.3 ± 6.9	0.915
**Sex**				
Male	46 (68.7 %)	28 (60.9 %)	18 (39.1 %)	0.161
Female	21 (31.3 %)	17 (81.0 %)	4 (19.0 %)	
**Comorbidities**				
None	16 (23.9 %)	13 (81.3 %)	3 (18.2 %)	0.343
1	30 (44.8 %)	18 (60 %)	12 (40 %)	
≥2	21 (31.4 %)	14 (66.7 %)	7 (33.3 %)	
**Diabetes mellitus**	21/62	12 (57.1 %)	9 (42.9 %)	
Yes	21	12	9	0.396
No	41	29	12	
**Cardiovascular diseases**	23/62	13 (56.5 %)	10 (43.5 %)	
Yes	23	13	10	0.271
No	39	28	11	
**Liver disease**	13/62	7 (53.8 %)	6 (46.2 %)	
Yes	13	7	6	0.334
No	49	34	15	
**Kidney disease**	5/62	4 (80 %)	1 (20 %)	
Yes	5	4	1	0.654
No	57	37	20	

More specifically, we analyzed the association between the presence of comorbidities, including diabetes mellitus, cardiovascular diseases, and liver and kidney diseases. The data presented in Table 2 show that in this cohort, no significant association was observed between the presence of comorbidity and COVID-19 mortality.

### 3.2 CRP levels were higher in non-surviving patients with COVID-19

**Fig. 1:**
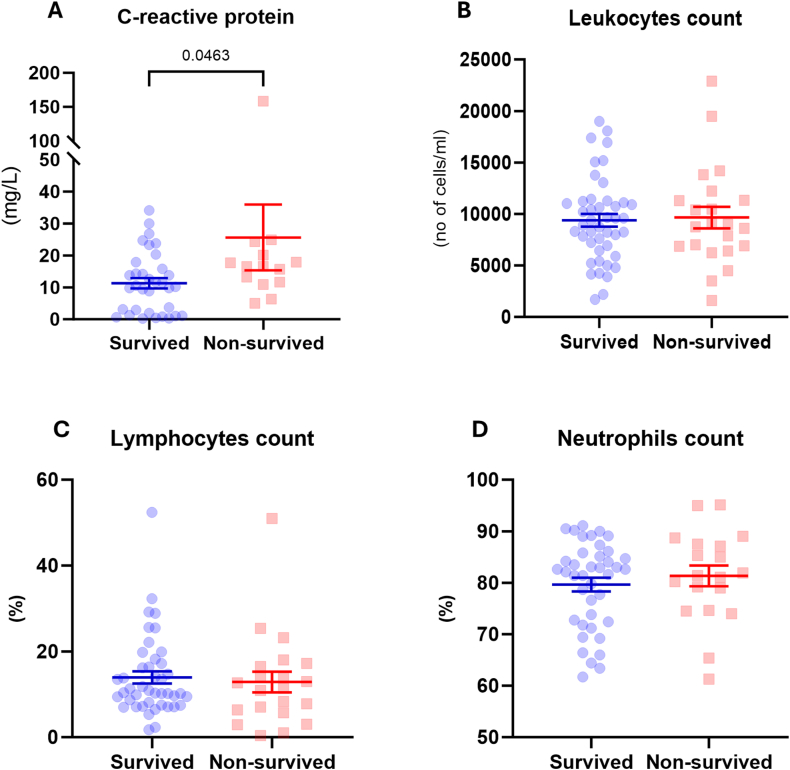
Analysis of inflammatory markers in the peripheral whole blood of patients with COVID-19. Blood samples were collected at the time of patients' admission and markers of systemic inflammation were analyzed. **A)** The levels of C-reactive protein were significantly higher in non-survived COVID-19 patients than the survived patients. However, there was no significant difference on **B**) Total leukocytes, **C)** Lymphocytes, and **D)** Neutrophils counts between survived and non-survived patients. (Survived group, n = 34–44; non-survived group, n = 14–22; numbers indicate P value).

Subsequently, we analyzed a marker of systemic inflammation, CRP, in non-survived patients compared with recovered patients. We observed a significantly higher serum CRP level in non-survived patients, indicating a higher level of systemic inflammation in these patients (Fig. 1A). However, there was no significant difference in the total leukocyte, lymphocyte, and neutrophil counts between the two groups (Fig. 1B–D).

### 3.3 ISGs expression was elevated in non-surviving patients with COVID-19

Interferon signaling plays a key role in regulating systemic inflammation in patients with COVID-19. To assess association of interferon stimulation with COVID-19 outcome, we examined the expression level of interferon-stimulated genes (ISGs) in total RNA isolated from the peripheral blood of these patients. We detected the expression of IFIT1, CH25H, viperin, ISG15, TRIM5, IFITM1, and MX1. All of these are ISGs [25] and can be used as markers for interferon type I-mediated stimulation.

We performed multivariate regression analysis to assess whether patients' characteristics, comorbidities and corticosteroid therapy affected ISGs expression. Results shown in Table 3 suggested that patients’ age, sex and the presence of comorbidities did not associate with any of the ISGs expressions. In keeping with this observation, corticosteroid therapy did not correlate with the expression of most of the ISGs, with the exception of TRIM5 (Table 3).

**Table 3:** Multivariate analysis of the association between patients’ demographic data, comorbidities, corticosteroid treatment and ISGs expression.

	IFIT1		CH25H		Viperin		ISG15		TRIM5		IFITM1		MX1	
	β	*P*	β	*P*	β	*P*	β	*P*	β	*P*	β	*P*	β	*P*
Age	−0.09	0.51	−0.13	0.36	−0.09	0.51	−0.09	0.54	−0.13	0.35	−0.06	0.69	−0.002	0.99
Sex	−0.18	0.18	−0.16	0.23	−0.08	0.54	0.06	0.65	−0.12	0.33	0.14	0.3	0.08	0.52
Diabetes	−0.10	0.46	−0.07	0.62	0.07	0.6	−0.19	0.16	0.02	0.87	−0.09	0.51	0.002	0.99
CV disease	0.09	0.55	0.15	0.32	0.12	0.42	−0.07	0.63	0.03	0.82	−0.01	0.97	0.13	−0.36
Liver disease	−0.17	0.24	−0.14	0.31	−0.16	0.25	−0.09	0.51	−0.2	0.15	−0.04	0.77	0.07	0.62
Kidney disease	−0.04	0.8	−0.03	0.81	−0.02	0.91	−0.04	0.76	−0.01	0.95	−0.12	0.37	−0.15	0.28
Corticosteroid therapy	0.11	0.42	0.17	0.23	0.25	0.07	0.12	0.39	0.3	**0.03**	0.08	0.57	0.21	0.13

CV, cardiovascular; β, standardized regression coefficient; *P,* P value.

**Fig. 2:**
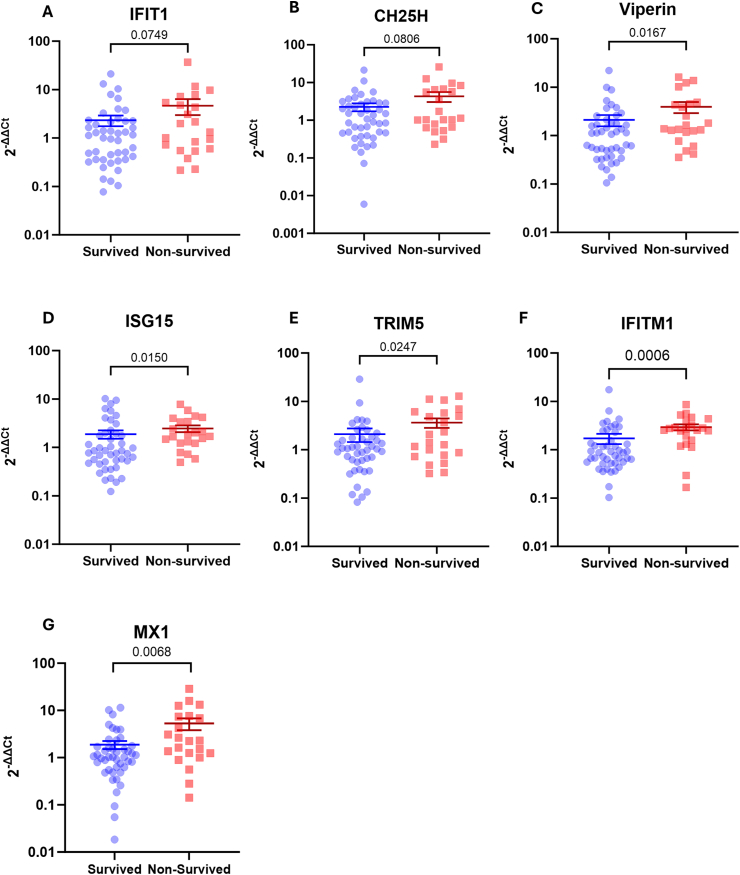
**Expressions of interferon-stimulated genes (ISGs) in patients with COVID-19.** Expressions of ISGs were analyzed on total RNAs isolated from peripheral whole blood. We found a trend of increase expression of **A)** Interferon-induced protein with tetratricopeptide repeats 1 (IFIT1) and **B)** cholesterol 25-hydroxylase (CH25H) in non-survived patients compared to survived group (P < 0.1). Furthermore, we observed statistically significant increase in the expressions of **C)** Viperin**, D)** interferon-stimulated gene 15 (ISG15), **E)** tripartite motif-containing protein 5 (TRIM15), **F)** interferon-induced transmembrane protein 1 (IFITM1), and **G)** MX dynamin like GTPase 1 (MX1) (Survived group, n = 45; non-survived group, n = 22; numbers indicate P values).

Next we analyzed ISGs levels comparing survived and non-survived patients. We found a strong trend of increased IFIT1 and CH25H expressions in non-survived patients with P values of approximately 0.07–0.08 (Fig. 2A and B). Moreover, the results shown in Fig. 2C–G demonstrate significant elevation in the expressions of other ISGs, including viperin, ISG15, TRIM, IFITM1, and MX1, in non-survived patients compared with that in recovered patients (P < 0.05). Overall, these findings suggest that a higher interferon signal might be associated with a poor prognosis and mortality in patients with moderate-to-severe COVID-19.

### 3.4 IFNAR1 and IFNAR2 expressions were elevated in non-survived patients

**Fig. 3:**
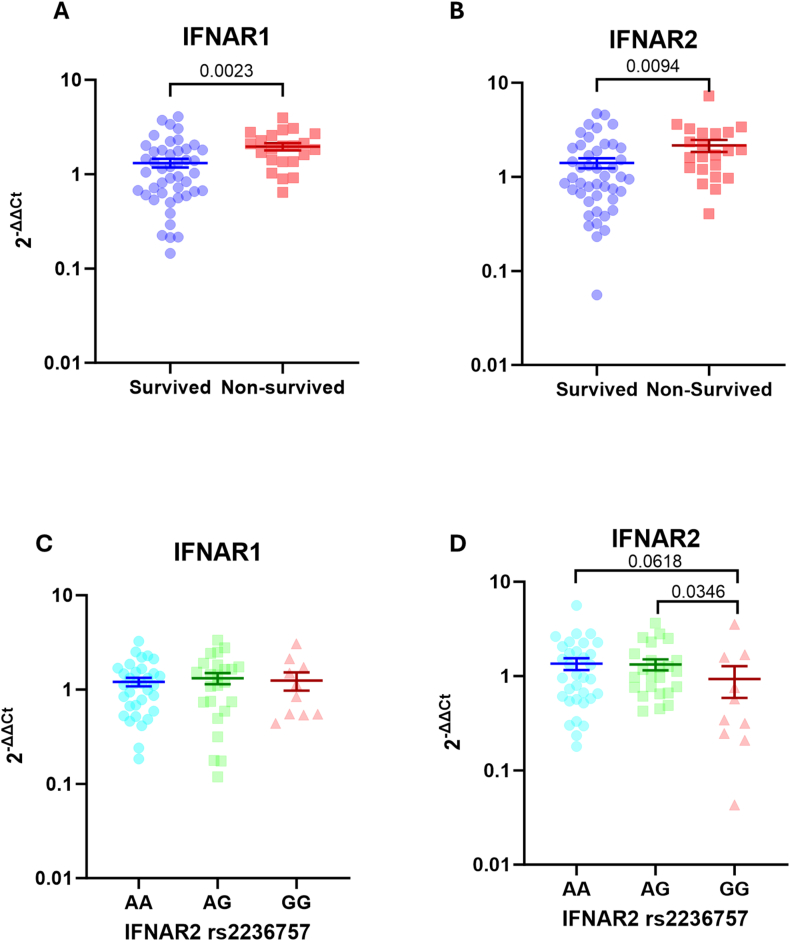
Expressions of interferon α/β receptor 1 (IFNAR1) and IFNAR2 according to patients' mortality and IFNAR2 rs2236757 SNP. There were significant increase in the expression of **A)** IFNAR1 and **B)** IFNAR2 in non-survived groups compared to survived patients (Survived group, n = 45; non-survived group, n = 22). The IFNAR2 rs2236757 SNP were detected in total DNA isolated from the peripheral whole blood using TaqMan assay. **C)** Our finding showed that there was no difference of IFNAR1 expression between individuals with different polymorphic alleles. **D)** However, we found that individuals with GG genotype of rs2236757 SNP exhibited lower level of IFNAR2 expression. (AA, n = 33; AG, n = 24; GG, n = 10, number indicate P value).

Interferon signaling is mainly mediated by interferon-α and interferon-β. The cellular response of these cytokines is mediated by the type-I interferon receptor, which are constituted by the interferon α/β receptor subunit 1 (IFNAR1) and subunit 2 (IFNAR2). Therefore, we examined the expression level of these IFNAR1 and IFNAR2 mRNAs in our patients and found that expression of both genes was significantly elevated in non-recovered patients (Fig. 3A and B).

### 3.5 *IFNAR2* gene polymorphism was associated with IFNAR2 expression but not with patient survival

The association between COVID-19 severity and interferon signaling was discovered in GWAS. One particular study has indicated that a common genetic variant within the *IFNAR2* gene (rs2236757) is strongly associated with COVID-19 severity [15]. Therefore, we examined the presence of the IFNAR2 rs2236757 SNP in our cohort and analyzed whether this genetic variation was correlated with IFNAR1 and IFNAR2 expression. As expected, we did not find any difference in the IFNAR1 expression level between the three IFNAR2 rs2236757 genotypes (Fig. 3C). In contrast, when assessing IFNAR2 mRNA expression according to the rs2236757 genotypes, we observed significantly lower IFNAR2 levels in individuals with the GG genotype than those with the AA and AG genotypes, whereas there was no difference in IFNAR2 level between patients with the AA and AG genotypes (Fig. 3D). These findings indicated the functionality of the IFNAR2 rs2236757 polymorphism in determining IFNAR2 but not IFNAR1 expression.

We then examined whether the IFNAR2 polymorphism was differently distributed according to the patients’ outcomes. The cross-tab analysis between the IFNAR2 polymorphism (rs2236757) and the survival of the COVID-19 group is presented in Table 4. We found no correlation between the IFNAR2 polymorphism and the survival of patients with COVID-19; however, the data showed a trend of higher frequencies of the A allele and AA genotype in non-survived compared to survived patients, although the difference did not reach statistical significance.

**Table 4:** Patients’ survival according to IFNAR2 rs2236757 genotypes.

	Survived	Non-survived
**IFNAR2 rs2236757 genotype**		
AA	20 (44.4 %)	13 (59.1 %)
AG	18 (40 %)	6 (27.3 %)
GG	7 (15.6 %)	3 (13.6 %)
P value = 0.506		
**IFNAR2 rs2236757 allele frequency**		
A	58 (64.4 %)	32 (72.7 %)
G	32 (35.6 %)	12 (27.3 %)
P value = 0.338		

### 3.6 IFNAR2 rs2236757 polymorphism did not affect ISGs expression

**Fig. 4:**
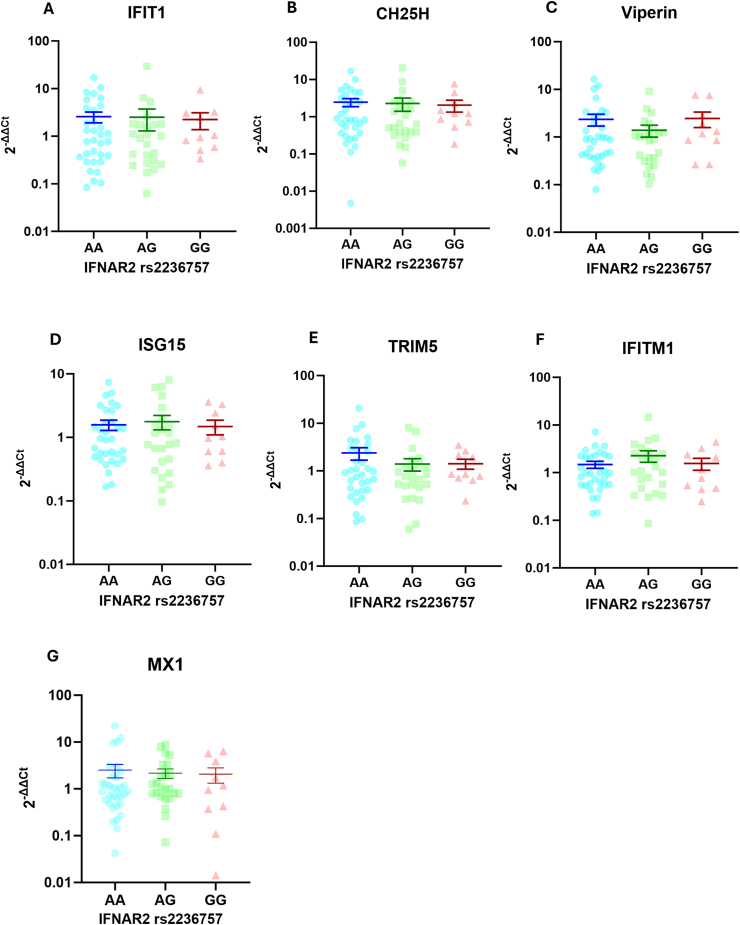
Comparison of ISGs expression between individuals with different IFNAR2 rs2236757 genotypes. We conducted analysis comparing the levels of ISGs expression between individuals with AA, AG and GG genotypes of the IFNAR2 rs2236757 SNP. We did not find any difference in the expressions of **A)** IFIT1, **B)** CH25H, **C)** Viperin, **D)** ISG15, **E)** TRIM5, **F)** IFITM1, and **G)** MX1, between subjects with AA, AG and GG genotype. (AA, n = 33; AG, n = 24; GG, n = 10).

To understand whether the rs2236757 genetic variant affects the expression of ISGs in the setting of COVID-19, we analyzed the expression of the six ISGs according to rs2236757. As shown in Fig. 4A–G, no difference in the expression of IFIT1, CH25H, viperin, ISG15, TRIM5, and IFIT1M was observed between patients with AA, AG, and GG genotypes. This indicates that despite causing reduced IFNAR2 expression, the rs2236757 polymorphism did not influence ISGs expression in patients with COVID-19.

### 3.7 IFNAR2 mRNA levels were not correlated with ISGs expression

To further evaluate whether IFNAR1 and IFNAR2 mRNA levels as well as IFNAR2 polymorphism were correlated with ISG expression levels in all patients, we performed multivariate regression analysis. As shown in Table 5, we did not observe any significant correlation between IFNAR1 and IFNAR2 mRNA with most of the ISGs tested; however, there was a significant association between IFNAR2 expression and viperin and MX1. We also observed significant association between IFNAR1 mRNA and IFITM1 level (Table 5). These data suggested that in our patient cohort, both IFNAR1 and IFNAR2 expression levels were not the main determinants of most ISGs expressions.

**Table 5:** Multivariable analysis of the association between IFNAR1 expression, IFNAR2 expression, IFNAR2 rs2236757 polymorphism and ISGs expression.

	IFIT1		CH25H		Viperin		ISG15		TRIM5		IFITM1		MX1	
	β	*P*	β	*P*	β	*P*	β	*P*	β	*P*	β	*P*	β	*P*
IFNAR1 mRNA	−0.04	0.80	−0.08	0.62	−0.20	0.21	−0.10	0.56	−0.11	0.50	0.52	**0.001**	0.07	0.63
IFNAR2 mRNA	0.07	0.67	0.12	0.50	0.43	**0.01**	0.20	0.24	0.27	0.11	−0.03	0.86	0.44	**0.004**
IFNAR2 polymorphism	−0.01	0.92	−0.02	0.85	0.02	0.90	0.03	0.81	−0.11	0.40	0.05	0.65	0.00	0.99

β, standardized regression coefficient; *P,* P value.

## 4 Discussion

In this study, we investigated the role of interferon-mediated signaling, a key determinant of the immune response in patients with COVID-19. Our main finding showed elevated ISGs expression levels in patients who did not survive COVID-19. This suggests an increased activation of interferon signaling in non-surviving patients, potentially linking it to the severity of the disease. However, our observation showed that this higher interferon signaling did not correlate with common polymorphism in the IFNAR2 gene or the levels of IFNAR2 expression.

The pivotal role of type I interferon signaling in orchestrating the immune response in COVID-19 is strongly supported by the finding that gene variations within the *IFNAR2* gene, which is responsible for encoding the receptor subunit, and TYK2, which encodes the downstream kinase of the receptor, exhibit significant associations with COVID-19 severity, as indicated by GWAS [15]. The study showed that low IFNAR2 expression and high TYK2 expression levels are linked to COVID-19 severity. This finding is consistent with other evidence showing that genetic defects associated with lower interferon type I signaling are associated with severe COVID-19 [17].

Contrary to expectations, we observed no significant difference in ISGs expression between patients with the GG genotype, which displayed lower IFNAR2 expression, and patients with other genotypes (AA and GA). Moreover, mortality rates exhibited no significant differences between individuals with the GG genotype and those with other genotypes associated with higher IFNAR2 expression. In addition, regression analysis indicated no association between IFNAR2 mRNA levels and most of ISGs expression levels. This finding underscores the complexity of the relationship between IFNAR2 genetics, interferon type 1 signaling, and ISGs expression. IFNAR2 and its downstream interferon type 1 pathway may not be the sole modulators of ISGs expression. Cho et al. reported the possibility of alternative pathways that control ISGs expression in the lung in the context of SARS-CoV-2 infection [26]. It is known that at least a subset of ISGs expressions may be regulated by IRF via a pathway that is independent of the JAK–STAT pathway (reviewed in Ref. [27]).

It is important to note that genetic background and ethnicity may influence the association between a genetic variant and phenotype. Studies assessing the replicability of GWAS between European and non-European ethnicities have revealed a significant lack of replication across different ethnic groups, underscoring the impact of genetic diversity on research outcomes [28]. Polymorphic allele frequency may also be varied in different populations. Interestingly, with regard to the IFNAR2 rs2236757 polymorphism, there were marked differences in the allele frequencies between different populations. Data from the NCBI database of SNP (dbSNP) suggested that the G allele appears to be the dominant allele in European and African populations, with a frequency of ∼70 %. In contrast, in the Asian populations, the G allele frequency is far less dominant (∼30–40 %). Our finding is consistent with these data, in which we found that the G allele frequency in our Indonesian cohort was 33 %.

Our finding that showed a trend of higher frequencies of the A allele and AA genotype of rs2236757 polymorphism in non-survived COVID-19 patients is in agreement with previous studies that were conducted in different ethnicities [[29], [30], [31]], indicating the critical role of this genetic variant in determining COVID-19 severity. Other studies also suggested an association between IFNAR2 rs2236757 and rs1051393 and susceptibility to infection with the omicron variant of the SARS-CoV-2 virus [32]. Moreover, there are several other polymorphic sites within the IFNAR2 gene that are important in COVID-19. The rs2834158, rs3153 and rs1051393 polymorphisms have been associated with the mortality of COVID-19 [31]. Other studies showed that rs2229207 and rs17860118 SNPs of the IFNAR2 gene increased the susceptibility to SARS-CoV-2 infection [33]. The other important polymorphism within the IFNAR2 gene is rs9984273. A report has suggested that the rs9984273 SNP was correlated with interferon-g and IL-6 expression in response to glucocorticoid treatment, which is logical given that this SNP is located in the binding motif of the glucocorticoid receptor [34]. Importantly, this SNP is also associated with COVID-19 severity and hospitalisation, and this SNP also contributes to the expression of IFNAR2 gene together with rs2236757 [34]. In essence, evidence is accumulating linking the IFNAR2 gene with the severity, mortality and susceptibility to COVID-19, indicating a crucial role of this gene in determining the course of this disease.

One key finding of our study is the finding that non-survived patients with COVID-19 displayed a higher ISGs expression level, which indicates elevated type I interferon signaling in individuals at the advanced stage of the disease and is consistent with previous reports [[35], [36], [37]]. This observation suggests that, in severe COVID-19 cases, therapeutic interventions aimed at inhibiting interferon 1 signaling could prove beneficial. The encouraging results from clinical trials using baricitinib, a TYK2 inhibitor, align with this concept [38,39]. However, as previously noted, reduced interferon signaling in the upper respiratory tract that occurs during the initial stages of the disease may compromise the early antiviral response, enabling the virus to evade innate immunity and infiltrate the lower respiratory tract. Therefore, interferon signaling induction during the initial stages of the disease, specifically targeting the upper respiratory tract, could be advantageous. This concept underpins the rationale for promising interferon therapy delivered as a nasal spray, focusing on the respiratory tract, which holds potential benefits in the early stages of COVID-19 [40].

Another implication arising from our study is the potential use of screening for ISGs expression as a predictive measure of COVID-19 severity. Elevated ISGs expression may serve as an indicative marker of excessive interferon type 1 signaling, prompting consideration for targeted treatment using interferon type 1 signaling inhibitors. Moreover, extending the assessment of ISGs expression to other viral diseases such as dengue fever and viral pneumonia may be essential for understanding the broader role of IFN type 1 signaling. This study may unveil possibilities to target this pathway in diverse diseases beyond COVID-19, fostering a more comprehensive understanding of its therapeutic potential.

We acknowledge several limitations of this study. The relatively small number of participants is the main limitation of this study. Further studies to expand the number and include patients from different ethnicities are important, thereby enhancing the study's external validity and generalizability. Our observation in this cohort showing that age, sex, and the presence of comorbidities did not determine survival, which is contrary to published data [41], might be due to the low number of participants. Another limitation is that our study only assessed gene expression at mRNA level. Investigating the expressions of IFNAR2, IFNAR1 and ISGs at protein level will deepen our understanding of the functional regulation of the interferon signaling pathway. In addition, our current analysis focused on a limited set of ISGs within the peripheral blood mononuclear cells of patients with COVID-19. A more extensive examination, potentially employing advanced techniques such as RNASeq, holds the promise of providing a more profound understanding of the intricate signaling pathways implicated in viral infections, particularly those induced by SARS-CoV-2.

In conclusion, our data add to the accumulating evidence on the crucial role of interferon type I signaling and ISGs expression in severe COVID-19 cases, despite a lack of association between the phenotypes and the common polymorphism in the *IFNAR2* gene. Therefore, to delineate the complexities between the IFNAR2 polymorphism, interferon 1 signaling, and ISGs expression, further studies are needed. Nevertheless, our findings may be useful in establishing novel ways to determine and/or predict COVID-19 severity as well as providing information about whether modulators of interferon type I signaling will be effective in controlling hyperinflammation in severe COVID-19.

## Funding source

This work was funded by a Grant from The 10.13039/100020300Institute for Research and Community Service (Lembaga Penelitian dan Pengabdian Masyarakat) 10.13039/501100008463Universitas Airlangga (Grant No 1031/UN3.15/PT/2021).

## Data availability statement

The datasets created and analyzed during this study are available from the corresponding author upon reasonable request. The data are not publicly available due to ethical restrictions.

## CRediT authorship contribution statement

**Berliana Hamidah:** Writing – original draft, Methodology, Investigation, Data curation. **Cennikon Pakpahan:** Writing – original draft, Investigation, Data curation. **Laksmi Wulandari:** Project administration, Data curation. **Damayanti Tinduh:** Supervision, Project administration. **Tri Wibawa:** Writing – review & editing. **Cita Rosita Sigit Prakoeswa:** Writing – review & editing, Supervision, Project administration, Conceptualization. **Delvac Oceandy:** Writing – review & editing, Writing – original draft, Supervision, Methodology, Formal analysis, Data curation, Conceptualization.

## Declaration of competing interest

The authors declare that they have no known competing financial interests or personal relationships that could have appeared to influence the work reported in this paper.

## References

[1] Stanifer M.L., Guo C., Doldan P., Boulant S. (2020). Importance of type I and III interferons at respiratory and intestinal barrier surfaces. Front. Immunol..

[2] Platanias L.C. (2005). Mechanisms of type-I- and type-II-interferon-mediated signalling. Nat. Rev. Immunol..

[3] Schoggins J.W. (2019). Interferon-stimulated genes: what do they all do?. Annu Rev Virol.

[4] Upasani V., Scagnolari C., Frasca F., Smith N., Bondet V., Vanderlinden A., Lay S., Auerswald H., Heng S., Laurent D., Ly S., Duong V., Antonelli G., Dussart P., Duffy D., Cantaert T. (2020). Decreased type I interferon production by plasmacytoid dendritic cells contributes to severe dengue. Front. Immunol..

[5] Shirasaki T., Honda M., Shimakami T., Murai K., Shiomoto T., Okada H., Takabatake R., Tokumaru A., Sakai Y., Yamashita T., Lemon S.M., Murakami S., Kaneko S. (2014). Impaired interferon signaling in chronic hepatitis C patients with advanced fibrosis via the transforming growth factor beta signaling pathway. Hepatology.

[6] Grajales-Reyes G.E., Colonna M. (2020). Interferon responses in viral pneumonias. Science.

[7] Queiroz M.A.F., Brito W., Pereira K.A.S., Pereira L.M.S., Amoras E., Lima S.S., Santos E.F.D., Costa F.P.D., Sarges K.M.L., Cantanhede M.H.D., Brito M., Silva A., Leite M.M., Viana M., Rodrigues F.B.B., Silva R.D., Viana G.M.R., Chaves T., Verissimo A.O.L., Carvalho M.D.S., Henriques D.F., Silva C.P.D., Nunes J.A.L., Costa I.B., Cayres-Vallinoto I.M.V., Brasil-Costa I., Quaresma J.A.S., Falcao L.F.M., Santos E., Vallinoto A.C.R. (2024). Severe COVID-19 and long COVID are associated with high expression of STING, cGAS and IFN-alpha. Sci. Rep..

[8] Sposito B., Broggi A., Pandolfi L., Crotta S., Clementi N., Ferrarese R., Sisti S., Criscuolo E., Spreafico R., Long J.M., Ambrosi A., Liu E., Frangipane V., Saracino L., Bozzini S., Marongiu L., Facchini F.A., Bottazzi A., Fossali T., Colombo R., Clementi M., Tagliabue E., Chou J., Pontiroli A.E., Meloni F., Wack A., Mancini N., Zanoni I. (2021). The interferon landscape along the respiratory tract impacts the severity of COVID-19. Cell.

[9] Del Valle D.M., Kim-Schulze S., Huang H.H., Beckmann N.D., Nirenberg S., Wang B., Lavin Y., Swartz T.H., Madduri D., Stock A., Marron T.U., Xie H., Patel M., Tuballes K., Van Oekelen O., Rahman A., Kovatch P., Aberg J.A., Schadt E., Jagannath S., Mazumdar M., Charney A.W., Firpo-Betancourt A., Mendu D.R., Jhang J., Reich D., Sigel K., Cordon-Cardo C., Feldmann M., Parekh S., Merad M., Gnjatic S. (2020). An inflammatory cytokine signature predicts COVID-19 severity and survival. Nat. Med..

[10] Fajgenbaum D.C., June C.H. (2020). Cytokine storm. N. Engl. J. Med..

[11] Tay M.Z., Poh C.M., Renia L., MacAry P.A., Ng L.F.P. (2020). The trinity of COVID-19: immunity, inflammation and intervention. Nat. Rev. Immunol..

[12] Blanco-Melo D., Nilsson-Payant B.E., Liu W.C., Uhl S., Hoagland D., Moller R., Jordan T.X., Oishi K., Panis M., Sachs D., Wang T.T., Schwartz R.E., Lim J.K., Albrecht R.A., tenOever B.R. (2020). Imbalanced host response to SARS-CoV-2 drives development of COVID-19. Cell.

[13] Broggi A., Ghosh S., Sposito B., Spreafico R., Balzarini F., Lo Cascio A., Clementi N., De Santis M., Mancini N., Granucci F., Zanoni I. (2020). Type III interferons disrupt the lung epithelial barrier upon viral recognition. Science.

[14] King C., Sprent J. (2021). Dual nature of type I interferons in SARS-CoV-2-induced inflammation. Trends Immunol..

[15] Pairo-Castineira E., Clohisey S., Klaric L., Bretherick A.D., Rawlik K., Pasko D., Walker S., Parkinson N., Fourman M.H., Russell C.D., Furniss J., Richmond A., Gountouna E., Wrobel N., Harrison D., Wang B., Wu Y., Meynert A., Griffiths F., Oosthuyzen W., Kousathanas A., Moutsianas L., Yang Z., Zhai R., Zheng C., Grimes G., Beale R., Millar J., Shih B., Keating S., Zechner M., Haley C., Porteous D.J., Hayward C., Yang J., Knight J., Summers C., Shankar-Hari M., Klenerman P., Turtle L., Ho A., Moore S.C., Hinds C., Horby P., Nichol A., Maslove D., Ling L., McAuley D., Montgomery H., Walsh T., Pereira A.C., Renieri A., Gen O.I., Investigators I.C., Initiative C.-H.G., andMe I., Investigators B., Gen C.I., Shen X., Ponting C.P., Fawkes A., Tenesa A., Caulfield M., Scott R., Rowan K., Murphy L., Openshaw P.J.M., Semple M.G., Law A., Vitart V., Wilson J.F., Baillie J.K. (2021). Genetic mechanisms of critical illness in COVID-19. Nature.

[16] Stark G.R., Kerr I.M., Williams B.R., Silverman R.H., Schreiber R.D. (1998). How cells respond to interferons. Annu. Rev. Biochem..

[17] Zhang Q., Bastard P., Liu Z., Le Pen J., Moncada-Velez M., Chen J., Ogishi M., Sabli I.K.D., Hodeib S., Korol C., Rosain J., Bilguvar K., Ye J., Bolze A., Bigio B., Yang R., Arias A.A., Zhou Q., Zhang Y., Onodi F., Korniotis S., Karpf L., Philippot Q., Chbihi M., Bonnet-Madin L., Dorgham K., Smith N., Schneider W.M., Razooky B.S., Hoffmann H.H., Michailidis E., Moens L., Han J.E., Lorenzo L., Bizien L., Meade P., Neehus A.L., Ugurbil A.C., Corneau A., Kerner G., Zhang P., Rapaport F., Seeleuthner Y., Manry J., Masson C., Schmitt Y., Schluter A., Le Voyer T., Khan T., Li J., Fellay J., Roussel L., Shahrooei M., Alosaimi M.F., Mansouri D., Al-Saud H., Al-Mulla F., Almourfi F., Al-Muhsen S.Z., Alsohime F., Al Turki S., Hasanato R., van de Beek D., Biondi A., Bettini L.R., D'Angio M., Bonfanti P., Imberti L., Sottini A., Paghera S., Quiros-Roldan E., Rossi C., Oler A.J., Tompkins M.F., Alba C., Vandernoot I., Goffard J.C., Smits G., Migeotte I., Haerynck F., Soler-Palacin P., Martin-Nalda A., Colobran R., Morange P.E., Keles S., Colkesen F., Ozcelik T., Yasar K.K., Senoglu S., Karabela S.N., Rodriguez-Gallego C., Novelli G., Hraiech S., Tandjaoui-Lambiotte Y., Duval X., Laouenan C., Clinicians C.-S., Clinicians C., Imagine C.G., French C.C.S.G., Co V.C.C., Amsterdam U.M.C.C.-B., Effort C.H.G., Group N.-U.T.C.I., Snow A.L., Dalgard C.L., Milner J.D., Vinh D.C., Mogensen T.H., Marr N., Spaan A.N., Boisson B., Boisson-Dupuis S., Bustamante J., Puel A., Ciancanelli M.J., Meyts I., Maniatis T., Soumelis V., Amara A., Nussenzweig M., Garcia-Sastre A., Krammer F., Pujol A., Duffy D., Lifton R.P., Zhang S.Y., Gorochov G., Beziat V., Jouanguy E., Sancho-Shimizu V., Rice C.M., Abel L., Notarangelo L.D., Cobat A., Su H.C., Casanova J.L. (2020). Inborn errors of type I IFN immunity in patients with life-threatening COVID-19. Science.

[18] Soltani-Zangbar M.S., Parhizkar F., Ghaedi E., Tarbiat A., Motavalli R., Alizadegan A., Aghebati-Maleki L., Rostamzadeh D., Yousefzadeh Y., Jadideslam G., Farid S.S., Roshangar L., Mahmoodpoor A., Heris J.A., Miahipour A., Yousefi M. (2022). A comprehensive evaluation of the immune system response and type-I Interferon signaling pathway in hospitalized COVID-19 patients. Cell Commun. Signal..

[19] World Health Organization (2020).

[20] Wulandari L., Hamidah B., Pakpahan C., Damayanti N.S., Kurniati N.D., Adiatmaja C.O., Wigianita M.R., Soedarsono, Husada D., Tinduh D., Prakoeswa C.R.S., Endaryanto A., Puspaningsih N.N.T., Mori Y., Lusida M.I., Shimizu K., Oceandy D. (2021). Initial study on TMPRSS2 p.Val160Met genetic variant in COVID-19 patients. Hum. Genom..

[21] Wang X., Spandidos A., Wang H., Seed B. (2012). PrimerBank: a PCR primer database for quantitative gene expression analysis, 2012 update. Nucleic Acids Res..

[22] Andersen C.L., Jensen J.L., Orntoft T.F. (2004). Normalization of real-time quantitative reverse transcription-PCR data: a model-based variance estimation approach to identify genes suited for normalization, applied to bladder and colon cancer data sets. Cancer Res..

[23] Livak K.J., Schmittgen T.D. (2001). Analysis of relative gene expression data using real-time quantitative PCR and the 2(-Delta Delta C(T)) Method. Methods.

[24] COVID-19 Treatment Guidelines Panel (2020). https://www.covid19treatmentguidelines.nih.gov/.

[25] Schoggins J.W., Rice C.M. (2011). Interferon-stimulated genes and their antiviral effector functions. Curr Opin Virol.

[26] Cho S.D., Shin H., Kim S., Kim H.J. (2023). Insights on interferon-independent induction of interferon-stimulated genes shaping the lung's response in early SARS-CoV-2 infection. Heliyon.

[27] Schneider W.M., Chevillotte M.D., Rice C.M. (2014). Interferon-stimulated genes: a complex web of host defenses. Annu. Rev. Immunol..

[28] Marigorta U.M., Navarro A. (2013). High trans-ethnic replicability of GWAS results implies common causal variants. PLoS Genet..

[29] Abdelhafez M., Nasereddin A., Shamma O.A., Abed R., Sinnokrot R., Marof O., Heif T., Erekat Z., Al-Jawabreh A., Ereqat S. (2023). Association of IFNAR2 rs2236757 and OAS3 rs10735079 polymorphisms with susceptibility to COVID-19 infection and severity in Palestine. Interdiscip Perspect Infect Dis.

[30] Dieter C., de Almeida Brondani L., Lemos N.E., Schaeffer A.F., Zanotto C., Ramos D.T., Girardi E., Pellenz F.M., Camargo J.L., Moresco K.S., da Silva L.L., Aubin M.R., de Oliveira M.S., Rech T.H., Canani L.H., Gerchman F., Leitao C.B., Crispim D. (2022). Polymorphisms in ACE1, TMPRSS2, IFIH1, IFNAR2, and TYK2 genes are associated with worse clinical outcomes in COVID-19. Genes.

[31] Fricke-Galindo I., Martinez-Morales A., Chavez-Galan L., Ocana-Guzman R., Buendia-Roldan I., Perez-Rubio G., Hernandez-Zenteno R.J., Veronica-Aguilar A., Alarcon-Dionet A., Aguilar-Duran H., Gutierrez-Perez I.A., Zaragoza-Garcia O., Alanis-Ponce J., Camarena A., Bautista-Becerril B., Nava-Quiroz K.J., Mejia M., Guzman-Guzman I.P., Falfan-Valencia R. (2022). IFNAR2 relevance in the clinical outcome of individuals with severe COVID-19. Front. Immunol..

[32] Skerenova M., Cibulka M., Dankova Z., Holubekova V., Kolkova Z., Lucansky V., Dvorska D., Kapinova A., Krivosova M., Petras M., Baranovicova E., Baranova I., Novakova E., Liptak P., Banovcin P., Bobcakova A., Rosolanka R., Janickova M., Stanclova A., Gaspar L., Caprnda M., Prosecky R., Labudova M., Gabbasov Z., Rodrigo L., Kruzliak P., Lasabova Z., Matakova T., Halasova E. (2024). Host genetic variants associated with COVID-19 reconsidered in a Slovak cohort. Adv. Med. Sci..

[33] Nhung V.P., Ton N.D., Ngoc T.T.B., Thuong M.T.H., Hai N.T.T., Oanh K.T.P., Hien L.T.T., Thach P.N., Hai N.V., Ha N.H. (2022). Host genetic risk factors associated with COVID-19 susceptibility and severity in Vietnamese. Genes.

[34] Jalkanen J., Khan S., Elima K., Huttunen T., Wang N., Hollmen M., Elo L.L., Jalkanen S. (2023). Polymorphism in interferon alpha/beta receptor contributes to glucocorticoid response and outcome of ARDS and COVID-19. Crit. Care.

[35] Lee J.S., Park S., Jeong H.W., Ahn J.Y., Choi S.J., Lee H., Choi B., Nam S.K., Sa M., Kwon J.S., Jeong S.J., Lee H.K., Park S.H., Park S.H., Choi J.Y., Kim S.H., Jung I., Shin E.C. (2020). Immunophenotyping of COVID-19 and influenza highlights the role of type I interferons in development of severe COVID-19. Sci Immunol.

[36] Wilk A.J., Rustagi A., Zhao N.Q., Roque J., Martinez-Colon G.J., McKechnie J.L., Ivison G.T., Ranganath T., Vergara R., Hollis T., Simpson L.J., Grant P., Subramanian A., Rogers A.J., Blish C.A. (2020). A single-cell atlas of the peripheral immune response in patients with severe COVID-19. Nat. Med..

[37] Zhou Z., Ren L., Zhang L., Zhong J., Xiao Y., Jia Z., Guo L., Yang J., Wang C., Jiang S., Yang D., Zhang G., Li H., Chen F., Xu Y., Chen M., Gao Z., Yang J., Dong J., Liu B., Zhang X., Wang W., He K., Jin Q., Li M., Wang J. (2020). Heightened innate immune responses in the respiratory tract of COVID-19 patients. Cell Host Microbe.

[38] Kalil A.C., Patterson T.F., Mehta A.K., Tomashek K.M., Wolfe C.R., Ghazaryan V., Marconi V.C., Ruiz-Palacios G.M., Hsieh L., Kline S., Tapson V., Iovine N.M., Jain M.K., Sweeney D.A., El Sahly H.M., Branche A.R., Regalado Pineda J., Lye D.C., Sandkovsky U., Luetkemeyer A.F., Cohen S.H., Finberg R.W., Jackson P.E.H., Taiwo B., Paules C.I., Arguinchona H., Erdmann N., Ahuja N., Frank M., Oh M.D., Kim E.S., Tan S.Y., Mularski R.A., Nielsen H., Ponce P.O., Taylor B.S., Larson L., Rouphael N.G., Saklawi Y., Cantos V.D., Ko E.R., Engemann J.J., Amin A.N., Watanabe M., Billings J., Elie M.C., Davey R.T., Burgess T.H., Ferreira J., Green M., Makowski M., Cardoso A., de Bono S., Bonnett T., Proschan M., Deye G.A., Dempsey W., Nayak S.U., Dodd L.E., Beigel J.H., Members A.-S.G. (2021). Baricitinib plus remdesivir for hospitalized adults with covid-19. N. Engl. J. Med..

[39] Marconi V.C., Ramanan A.V., de Bono S., Kartman C.E., Krishnan V., Liao R., Piruzeli M.L.B., Goldman J.D., Alatorre-Alexander J., de Cassia Pellegrini R., Estrada V., Som M., Cardoso A., Chakladar S., Crowe B., Reis P., Zhang X., Adams D.H., Ely E.W., Group C.-B.S. (2021). Efficacy and safety of baricitinib for the treatment of hospitalised adults with COVID-19 (COV-BARRIER): a randomised, double-blind, parallel-group, placebo-controlled phase 3 trial. Lancet Respir. Med..

[40] Fu W., Liu Y., Xia L., Li M., Song Z., Hu H., Yang Z., Wang L., Cheng X., Wang M., Jiang R., Liu L., Mao X., Chen J., Ling Y., Zhang L., Yan J., Shan F., Steinhart C., Zhang X., Zhu T., Xu J., Lu H. (2020). A clinical pilot study on the safety and efficacy of aerosol inhalation treatment of IFN-kappa plus TFF2 in patients with moderate COVID-19. EClinicalMedicine.

[41] Docherty A.B., Harrison E.M., Green C.A., Hardwick H.E., Pius R., Norman L., Holden K.A., Read J.M., Dondelinger F., Carson G., Merson L., Lee J., Plotkin D., Sigfrid L., Halpin S., Jackson C., Gamble C., Horby P.W., Nguyen-Van-Tam J.S., Ho A., Russell C.D., Dunning J., Openshaw P.J., Baillie J.K., Semple M.G., investigators I.C. (2020). Features of 20 133 UK patients in hospital with covid-19 using the ISARIC WHO Clinical Characterisation Protocol: prospective observational cohort study. BMJ.

